# Performance Analysis of Six Semi-Automated Tumour Delineation Methods on [^18^F] Fluorodeoxyglucose Positron Emission Tomography/Computed Tomography (FDG PET/CT) in Patients with Head and Neck Cancer

**DOI:** 10.3390/s23187952

**Published:** 2023-09-18

**Authors:** Francesco Bianconi, Roberto Salis, Mario Luca Fravolini, Muhammad Usama Khan, Matteo Minestrini, Luca Filippi, Andrea Marongiu, Susanna Nuvoli, Angela Spanu, Barbara Palumbo

**Affiliations:** 1 Department of Engineering, Università degli Studi di Perugia, Via Goffredo Duranti 93, 06125 Perugia, Italy; mario.fravolini@unipg.it (M.L.F.); muhammadusama.khan@studenti.unipg.it (M.U.K.); 2 Unit of Nuclear Medicine, Department of Medicine, Surgery and Pharmacy, University of Sassari, 07100 Sassari, Italy; r.salis15@studenti.uniss.it (R.S.); amarongiu2@uniss.it (A.M.); snuvoli@uniss.it (S.N.);; 3 Section of Nuclear Medicine and Health Physics, Department of Medicine and Surgery, Università degli Studi di Perugia, Piazza Lucio Severi 1, 06132 Perugia, Italy; matteo.minestrini@ospedale.perugia.it (M.M.); barbara.palumbo@unipg.it (B.P.); 4 Policlinico Tor Vergata Hospital, Viale Oxford 81, 00133 Rome, Italy; luca.filippi@ptvonline.it

**Keywords:** head and neck cancer, positron emission tomography, segmentation, region of interest, radiomics

## Abstract

*Background.* Head and neck cancer (HNC) is the seventh most common neoplastic disorder at the global level. Contouring HNC lesions on [${}^{18}$F] Fluorodeoxyglucose positron emission tomography/computed tomography (FDG PET/CT) scans plays a fundamental role for diagnosis, risk assessment, radiotherapy planning and post-treatment evaluation. However, manual contouring is a lengthy and tedious procedure which requires significant effort from the clinician. *Methods.* We evaluated the performance of six hand-crafted, training-free methods (four threshold-based, two algorithm-based) for the semi-automated delineation of HNC lesions on FDG PET/CT. This study was carried out on a single-centre population of n=103 subjects, and the standard of reference was manual segmentation generated by nuclear medicine specialists. Figures of merit were the Sørensen–Dice coefficient (DSC) and relative volume difference (RVD). *Results.* Median DSC ranged between 0.595 and 0.792, median RVD between −22.0% and 87.4%. Click and draw and Nestle’s methods achieved the best segmentation accuracy (median DSC, respectively, 0.792 ± 0.178 and 0.762 ± 0.107; median RVD, respectively, −21.6% ± 1270.8% and −32.7% ± 40.0%) and outperformed the other methods by a significant margin. Nestle’s method also resulted in a lower dispersion of the data, hence showing stronger inter-patient stability. The accuracy of the two best methods was in agreement with the most recent state-of-the art results. *Conclusions.* Semi-automated PET delineation methods show potential to assist clinicians in the segmentation of HNC lesions on FDG PET/CT images, although manual refinement may sometimes be needed to obtain clinically acceptable ROIs.

## 1. Introduction

Head and neck cancer (HNC) is the seventh most common oncological disorder worldwide with over 660,000 new cases and 325,000 related deaths per year [1]. Current estimates place the four-year survival rate after diagnosis at 72% for men and 76% for women [2].

The primary prevention of HNC involves reducing the exposure to risk factors such as tobacco smoking, alcohol consumption, human papillomavirus (HPV) and Epstein–Barr virus (EBV) infections. Secondary prevention could also give excellent results due to the good prognosis when the disease is diagnosed at an early stage [3,4]. In this context, positron emission tomography/computed tomography with [${}^{18}$F] Fluorodeoxyglucose (FDG PET/CT in the remainder) is currently one of the most reliable methods for the management of patients with HNC, its role being crucial for the diagnosis of the primary lesion, staging (detection of loco-regional lymph-node metastasis and distant metastasis) and follow-up after therapy [5,6]. However, a qualitative analysis of FDG PET/CT images is not always sufficient to obtain correct diagnosis and/or treatment monitoring. Quantitative data are necessary to better define the staging and re-staging of the disease.

Advances in technology and the advent of radiomics in nuclear medicine have the potential to obtain the accurate quantification of data extracted from images, thus providing non-invasive, personalised modelling, treatment planning and response assessment. The aim of radiomics is to convert medical images into mineable data that can be used for computer-assisted clinical decision making [7]. Radiomics relies on the ability to detect clinically relevant, sub-visual features that would go unnoticed to the human eye [8,9]. It also leverages on artificial intelligence methods and large datasets of pre-classified cases to make predictions about, for instance, disease phenotypes, or survival and response to therapy.

There are two main branches of radiomics: *conventional* (or *hand-crafted*) and based on *deep learning* [10]. The main difference is that image features are defined a priori in conventional radiomics, whereas, in deep learning, they are learnt from data. Both approaches have their pros and cons, and it is currently under debate which is the most suitable for translation into the clinical practice [11]. Although deep learning radiomics may produce more accurate prediction models, the gain comes at a cost—i.e., large datasets and computational resources needed for training, and the limited interpretability of the models (the ‘black box’ problem). On the other hand, conventional radiomics is computationally less expensive and relies on relatively easy-to-interpret image features which can be directly linked to the underlying characteristics of the tissue [12]. The existence of various, easy-to-use radiomics tools based on conventional radiomics [13] also facilitates testing and application in clinical environments. The above pros and cons translate seamlessly into lesion delineation (more on this later). Although methods based on deep learning may achieve better performance in some cases, they require large datasets for training and highly skilled personnel for model set-up and tuning. On the other hand, conventional methods are essentially training-free, easy to interpret and user-friendly.

Conventional radiomics (the focus of this work) comprises six steps [14,15]: acquisition, pre-processing, segmentation, feature extraction, post-processing and data analysis. Among them, the segmentation and feature extraction step are crucial. The first the identification and delineation of the region(s) of interest (ROI), the second involves the computation of sets of pre-defined (hand-crafted) image parameters such as first-order statistics, shape and texture features from the ROI.

In recent years, conventional radiomics applied to FDG PET/CT has demonstrated its potential to assist clinical decision making in patients with head and neck cancer [16]. Recent studies have supported the use of FDG PET/CT radiomics for predicting overall survival [17,18,19,20], disease-free survival [17,18,19,21], metastasis-free survival [19], loco-regional recurrence [22], local control [23] and response to therapy [24]. The outcome of radiomics studies, however, can be affected by a number of factors including study design (e.g., perspective vs. retrospective); image acquisition and reconstruction settings; spatial resampling; lesion delineation; signal quantisation and others. Such sources of uncertainty may easily lead to models that fail to generalise to new research trials [16,25,26]. Lesion delineation (also referred to as *segmentation* or *contouring*), in particular, is a critical step in the radiomics process, as the correct identification of the ROI is crucial to the development of robust prediction models.

Contouring suspicious head and neck lesions poses specific challenges due to the intrinsic complexity of the anatomical region. Manual delineation performed by experienced physicians is usually regarded as the standard of reference. Unfortunately, this is a tedious and time-consuming procedure, and, as such, can represent a significant bottleneck to the whole procedure. To overcome this problem, a number of automated and semi-automated approaches have been proposed in the literature, some of which are already available in radiomics tools.

Although comparing different methods for lesion segmentation has been the subject of a number previous studies—particularly in lung cancer [27,28]—the delineation of HNC lesions on FDG PET/CT has received much less attention in the literature. Prior related works include a paper by Zaidi et al. [29], in which image segmentation techniques were compared against the reference specimens of pharyngolaryngeal squamous cell carcinoma collected ‘en bloc’ and frozen—although the analysis was essentially two-dimensional as the specimens were cut into 1.7–2 mm thick slices. More recently, Trada et al. [30] assessed the impact of tree semi-automated tumour delineation methods on the stability of semi-quantitative parameters computed from FDG PET/CT, but no direct assessment of ROI overlap was carried out. Other works have advocated for the use of convolutional network for segmentation [31], but these are outside the scope of this study as we focus on ready-to-use, training-free methods.

In this work, we benchmarked six semi-automated, training-free methods for segmenting HNC lesions on FDG PET/CT. The analysis was carried out on a retrospective, single-centre patient population using LIFEx, a freeware tool for radiomic feature calculation. The Sørensen–Dice coefficient and relative volume difference were, respectively, the primary and secondary figure of merit for the segmentation goodness. The standard of reference was manual delineation operated by nuclear medicine physicians. To the best of our knowledge, this is the first study of this kind in head and neck cancer; a disease that requires particular attention due to its poor prognosis and the heterogeneous characteristics of the anatomical district.

## 2. Materials and Methods

### 2.1. Patient Population

For this study, we retrospectively evaluated *n* = 103 head and neck lesions (volume = 22.7 ± 27.8 [0.5–157.7 cm${}^{3}$]) from as many subjects (male = 79, female = 24; age = 63.2 ± 9.2 (42–88 years)) who received baseline FDG PET/CT scans for clinical examination at the Unit of Nuclear Medicine of Department of Medicine, Surgery and Pharmacy; Università degli Studi di Sassari, Sassari, Italy, between December 2014 and March 2023. The inclusion criteria were:

- Age > 18;
- No previous surgical, chemotherapy and/or radiotherapy treatment for the suspicious lesion before the FDG PET/CT scan was obtained;
- Presence of a histology record for the region of interest after surgical resection.

All lesions were subsequently confirmed as head and neck squamous cell carcinomas (HNSCCs) by histological evaluation. Only primary lesions were considered for the analysis, and therefore, lymph-nodes and secondary lesions were not included in this study. The anatomical site distribution was: Oral cavity/oropharynx (n=71), laringopharynx (n=22), nasopharynx (n=7), and multiple sites (n=3).

### 2.2. Image Acquisition

The scans were performed according to the AIMN guidelines [32] on a Discovery 710 PET/CT machine (GE HealthCare Technologies Inc., Chicago, IL, United States). Patients were required to fast for at least 6 h prior to the procedure and checked for glucose level lower than 150 mg/dL before the examination; then, image acquisition started 60 min after radiotracer injection. The PET images of matrix size 256 px × 256 px, in-plane pixel spacing 2.73 mm × 2.73 mm and slice thickness 3.27 mm were reconstructed by ordered subset expectation maximisation with point spread function recovery and time-of-flight (VPFXS). The dimension of the field of view of the PET camera was 700 × 153 mm. Low-dose CT scans for attenuation correction and anatomical correlation were acquired in helicoidal mode under 120–140 kVp tube voltage and reconstructed into images of size of 512 px × 512 px, in-plane pixel spacing of 1.37 mm × 1.37 mm, slice thickness of 3.75 mm and spacing between slices of 3.27 mm. The total number of slices for patient ranged from 83 to 551 for PET and from 83 to 558 for CT. No pre-processing step like filtering, resampling or spatial rescaling was applied to the resulting images.

### 2.3. Lesion Delineation

**Figure 1:**
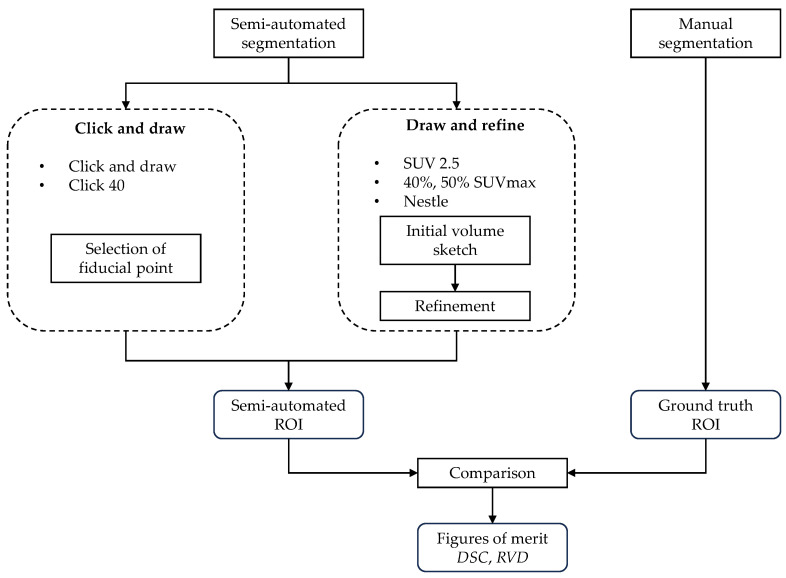
Experimental set-up: Summary flow-chart.

**Figure 2:**
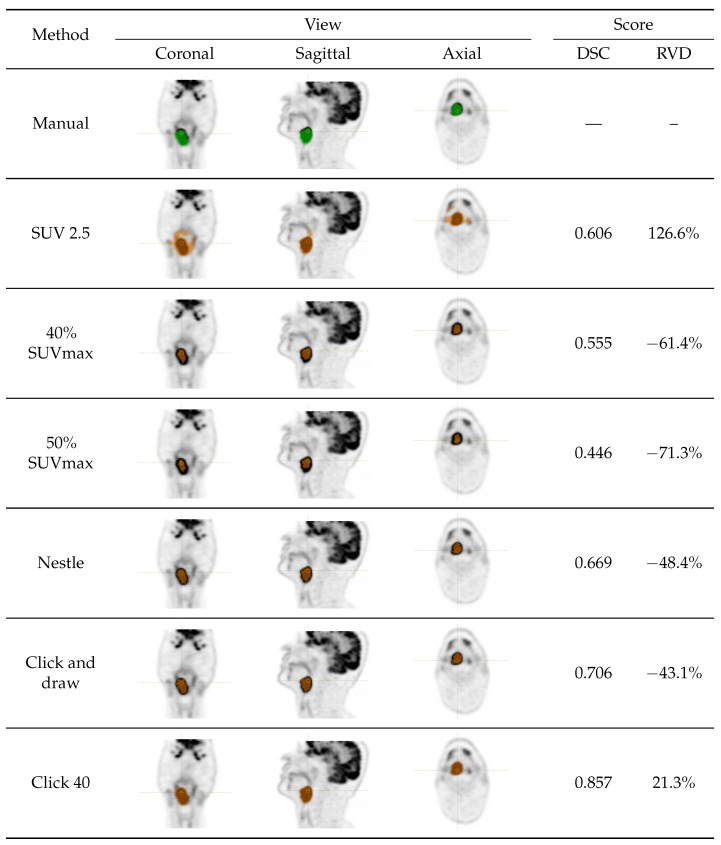
Squamous cell carcinoma of the oropharynx in a 56-year-old male (0d64). Rows refer to the segmentation method used with manual segmentation on the top row. The columns Coronal, Sagittal and Axial report snapshots of the obtained segmentation on the three planes. Note that green overlays denote manual (ground truth) segmentation and brown overlays the semi-automatically generated ROIs. Finally, the last two columns on the right report the figures of merit (DSC, RVD) that each semi-automated segmentation method achieved in this case. Observe the spillover effect that affects SUV 2.5 and which leads to a markedly overestimated volume.

**Figure 3:**
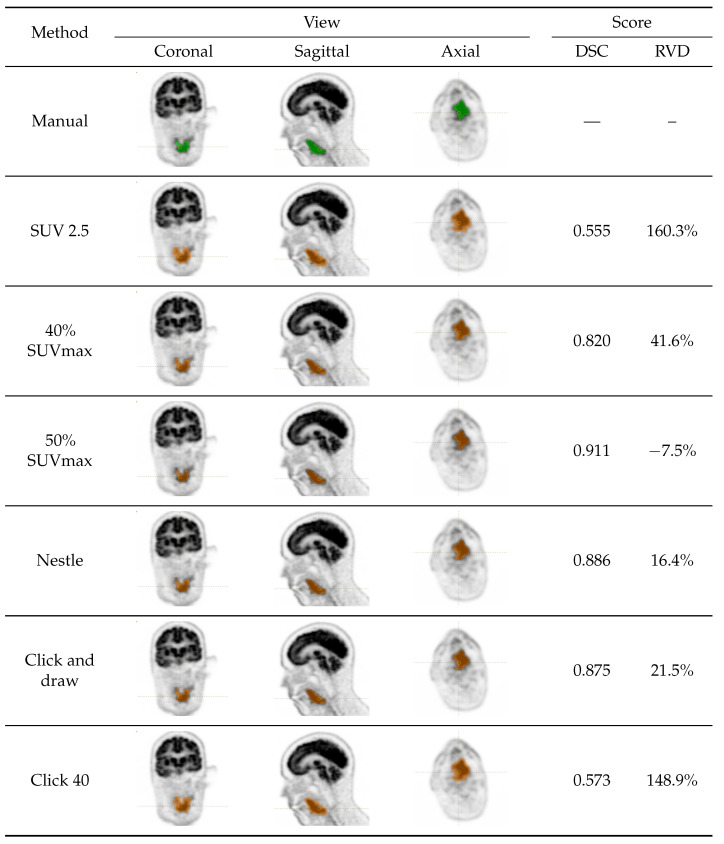
Squamous cell carcinoma of the oropharynx in a 66-year-old male (76b9). Rows refer to the segmentation method used with manual segmentation on the top row. The columns Coronal, Sagittal and Axial report snapshots of the obtained segmentation on the three planes. Note that green overlays denote manual (ground truth) segmentation and brown overlays denote the semi-automatically generated ROIs. Finally, the last two columns on the right report the figures of merit (DSC, RVD) that each semi-automated segmentation method achieved in this case. Like in Figure 2, we note that the spillover effect is again evident on SUV 2.5, but Click 40 also produces a patently overestimated volume in this case.

Six semi-automated, training-free lesion delineation methods of the FDG PET signal were considered in this study (Section 2.3.1, Section 2.3.2, Section 2.3.3, Section 2.3.4, Section 2.3.5 and Section 2.3.6) and compared with manually generated ground-truth segmentation (Section 2.3.7). Specifically, we evaluated one-fixed absolute threshold method, two fixed relative threshold methods, one background threshold method and two algorithm-based methods (see Table 1 for a recap; the overall experimental set-up is also summarised in Figure 1). All the semi-automated and manual delineation procedures were carried out using LIFEx v7.3.0 (LITO-Curie, SHFJ-CEA, CNRS, Univ. Paris Sud, University Paris Saclay, Orsay, France [33]). Figure 2 and Figure 3 show the ground-truth and the segmentation results for two sample cases.

#### 2.3.1. Absolute Threshold at SUV 2.5 (SUV 2.5)

Absolute thresholding is one of the simplest and most widespread method for FDG PET/CT image segmentation. It consists of setting a threshold value to separate the lesion from the background, and in this case, we set the threshold at 2.5 of the standardised uptake value (SUV), which is the typical choice in the literature [29]. As a result, all voxels with an SUV above the threshold are assigned to the ROI.

Operatively, we can define this as a ‘draw and refine’ procedure (Table 1, Figure 1), which involves two steps. In the first, the user sketches an initial three-dimensional volume that completely encircles the putative ROI; then all the voxels with uptake below the threshold are filtered out of the initial volume. We used a three-dimensional spherical painting tool for sketching the initial volume, and the physician was free to adjust the radius and position of the sphere(s) during this operation.

#### 2.3.2. Relative Threshold at 40% of SUVmax (40% SUVmax)

Relative thresholding is also a very common approach for lesion delineation on FDG PET/CT. Operatively, this is analogous to SUV 2.5 (draw and refine); however, in this case, the threshold is defined as a percentage (40%) of the maximum SUV in the initial volume.

#### 2.3.3. Relative Threshold at 50% of SUVmax (50% SUVmax)

Same as 40% SUVmax (Section 2.3.2), but with the threshold set at 50% of SUVmax. Note that we also performed preliminary tests with other threshold values (30% and 70%), but discarded these options as the results were not satisfactory.

#### 2.3.4. Nestle’s Method (Nestle)

This technique is also based on relative thresholding, and is therefore conceptually similar to 40% SUVmax and 50% SUVmax (Section 2.3.2 and Section 2.3.3). The calculation of the threshold, however, is more involved in this case as it takes into account not only the radiotracer uptake of the initial region, but also that of the background. The threshold is computed as follows: [36]:

(1) $$t_{N}=\beta \times I_{70}+I_{bgd}$$

where $I_{70}$ indicates the mean uptake value in a volume containing all the voxels with an uptake of >70% of the maximum value (70% $I_{max}$ isocontour volume), and $I_{bgd}$ denoting the average background uptake. The latter is defined as the mean uptake in a shell of 4 mm thickness generated by offsetting the 70% SUVmax iso-contour boundary towards the exterior by 12 mm. Voxels in the shell with an uptake greater than SUV 2.5 units are excluded from the calculation. Finally, β is a tuning parameter which depends on the acquisition device, the acquisition settings and the reconstruction settings. The value of β should in principle be estimated through phantom experiments. Since no phantom data were available for this study, we preliminarily tested β=0.15 and β=0.30 (as, respectively, proposed in [36,38]) and chose the latter as it provided better qualitative results. Further details about this method are also available in Ref. [37].

#### 2.3.5. Click and Draw

This is an algorithm-based method of the type ‘click and draw’ (Table 1, Figure 1). The only interaction required from the user is the selection of a point approximately around the centroid of the region to be delineated.

Let $V_{s}$ indicate the voxel corresponding to the selected point, and denote by $I(V_{s})$ the signal intensity (tissue uptake) at that voxel. The algorithm proceeds as follows (see also [37] for further details):

1. Perform a three-dimensional flood-fill [39] that extends to all voxels connected to $V_{s}$ with an uptake of at least $t\times I(V_{s})$, where t=0.7. Let Ω denote the resulting (flood-filled) region.
  - Return an exception if $|\Omega |>500\;{\mathrm{cm}}^{2}$, where |x| indicates the extent (volume in this case) of *x*.
2. Let $I_{N}$ be Nestle’s uptake threshold computed via Nestle’s method (see Section 2.3.4) on |Ω| using β=0.3 and $I_{\max}$ as the input parameters, where Imax=maxV∈Ω[I(V)].
3. Perform a three-dimensional flood-fill again, this time starting at VM=argmaxV∈Ω[I(V)], and including all voxels connected to $V_{M}$ with an uptake of at least $I_{N}$.
4. Obtain the flood-filled region as the resulting ROI.

#### 2.3.6. Click 40

This method is based on the same algorithm as Click and draw (Section 2.3.5) with the only difference being that a threshold value t=0.4 instead of t=0.7 is used for step 1.

#### 2.3.7. Ground Truth

Reference (ground-truth) segmentation of the PET signal was generated via manual, slice-by-slice contouring on transaxial slices of the fused PET/CT images. The contouring was carried out by a nuclear medicine resident (R.S., >3 years experience) under the supervision of a senior nuclear medicine specialist (B.P., >20 years experience). For the process, the operators used a ‘CTL-472 One’ graphics tablet (Wacom Co., Ltd., Saitama, Japan) and were free to adjust the size of the painting tool and the image magnification factor based on their experience and personal judgement.

### 2.4. Evaluation Metrics

The effectiveness of the semi-automated lesion delineation methods described in Section 2.3.1, Section 2.3.2, Section 2.3.3, Section 2.3.4, Section 2.3.5 and Section 2.3.6 was assessed by comparing the results of each method against manual ground-truth segmentation. We considered the Sørensen–Dice coefficient (DSC) and relative volume difference (RVD) as the primary and secondary figures of merit [40,41,42]:

(2) $$\mathrm{DSC}=\frac{2\left|\Omega_{e}\cap \Omega_{gt}\right|}{\left|\Omega_{e}\right|+\left|\Omega_{gt}\right|}$$

(3) $$\mathrm{RVD}=\frac{\left|\Omega_{e}\right|-\left|\Omega_{gt}\right|}{\left|\Omega_{gt}\right|}$$

In Equations (2) and (3), $\Omega_{e}$ and $\Omega_{gt}$, respectively, denote the result of semi-automated segmentation and the ground truth ROI.

Let us recall that the Sørensen–Dice coefficient measures the overlap between two sets, and is arguably the most used metric for validating image segmentation algorithms. It ranges from 0 to 1, with 1 indicating perfect overlap (i.e., perfect segmentation) and 0 indicating no overlap [43,44]. Relative volume difference quantifies the extent to which the volume of semi-automated segmentation differs from that of the ground-truth ROI [40,41]. A positive RVD indicates that the algorithm overestimates the target volume, and a negative RVD indicates that it underestimates it. Note that the relative volume difference should never be considered alone, but always in conjunction with DSC or other metrics, as RVD can be zero even when there is no overlap between the estimated and the target ROI.

### 2.5. Statistical Analysis

Statistical differences between the performance of the segmentation methods were assessed via pairwise non-parametric Mann–Whitney U test at a significance value α=0.05. Bonferroni’s correction for multiple tests was also applied.

## 3. Results

### 3.1. Segmentation Overlap Assessed by Sørensen–Dice Coefficient

**Figure 4:**
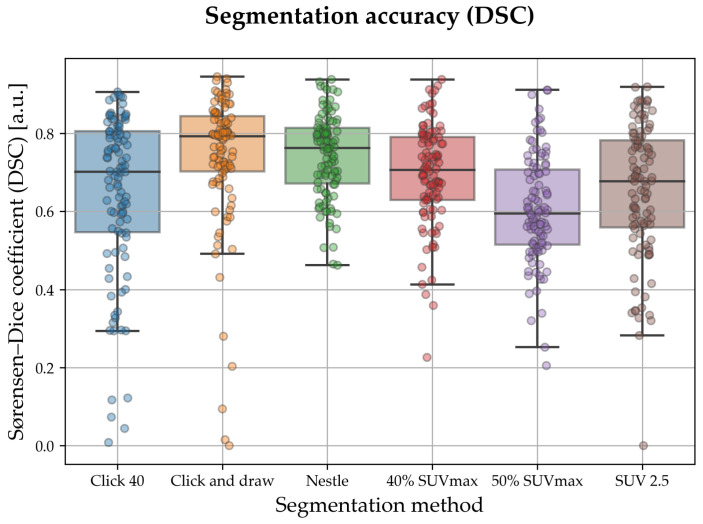
Box plots/strip plots showing DSC values (Sørensen–Dice coefficient) for the semi-automated segmentation methods considered in this study. Colour and *x* tick labels denote segmentation method; each dot in the strip plots represents one lesion/patient. Observe that click and draw achieved the highest median DSC followed by Nestle; the latter, however, had a remarkably lower dispersion of the data.

The box plots/strip plots in Figure 4 show the distribution of the Sørensen–Dice coefficient (DSC) for the semi-automated methods considered in this study. Aggregate values are also reported in Table 2.

We observe that, in absolute terms, the two best methods (click and draw and Nestle), respectively, achieved an aggregate DSC of 0.792 and 0.762, figures which are in good agreement (and even slightly higher) than has been reported in the recent literature [21,31]. Figure 4 also shows a marked dispersion of the data, which indicates strong inter-case variability. This was particularly evident for click and draw and click 40, which sometimes resulted in a DSC close to zero, indicating a completely misplaced ROI. By contrast, Nestle had the lowest DSC spread (assessed by standard deviation) among the methods considered.

On a relative scale, we observe that click and draw and Nestle outperformed the other methods by a noticeable margin in terms of median DSC. Pairwise comparison by Mann–Whitney U test (Table 3) indicates that there was no statistically significant difference between these two methods. However, click and draw had a significantly higher DSC score than all other methods except Nestle, while Nestle significantly outperformed SUV 2.5 and 50% SUVmax.

### 3.2. Relative Volume Difference

**Figure 5:**
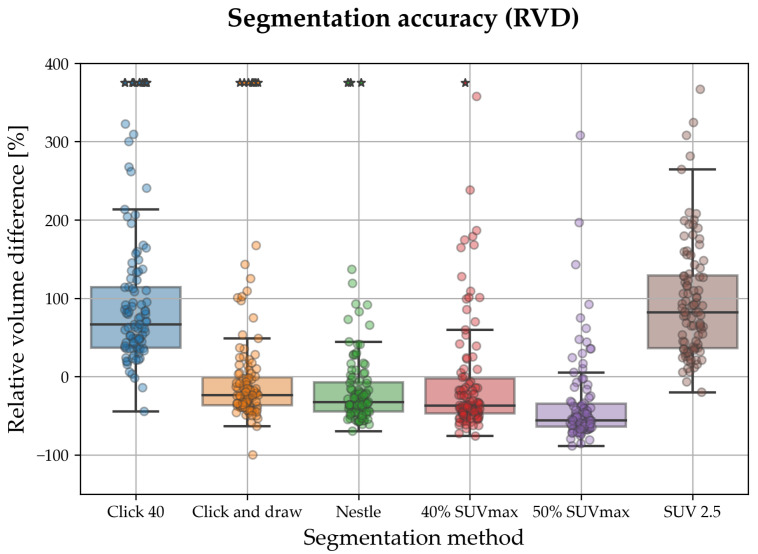
Box plots/strip plots showing relative volume difference (RVD) for the semi-automated segmentation methods considered in this study. Colour and *x* tick labels denote segmentation method; each dot in the strip plots represents one lesion/patient, stars indicate outliers (plotted out of scale). Note that all the methods except click 40 and SUV 2.5 tended to underestimate the lesion volume; on the whole, click and draw and Nestle had the lowest RVD magnitude.

Relative volume difference (Figure 5, Table 2) indicating that all methods except Click 40 and SUV 2.5 had a tendency to underestimate the lesion volume. On the whole, click and draw and Nestle had the lowest RVD magnitude (respectively, −21.6% and −32.7%); SUV 2.5 the highest (89.9%). Pairwise RVD comparison (Table 3) highlighted significant differences between all pairs except between Click and draw and Nestle, Click 40 and SUV 2.5, and Nestle and 40% SUVmax. We again observe that Nestle had the lowest standard deviation (i.e., highest inter-patient stability) in terms of RVD than the other methods; click 40 and click and draw the highest variability.

### 3.3. Time Savings

A quantitative evaluation of the time required for manual segmentation, semi-automated ‘click and draw’ segmentation and semi-automated ‘draw and refine’ segmentation (see also Table 1 for a recap) were also carried out on a sample of five cases. The results are reported in Table 4. As can be seen, the time saving achieved by semi-automated methods was remarkable.

## 4. Discussion

Head and neck tumour volume delineation on FDG PET/CT plays a pivotal role in clinical decisions such as risk stratification, prediction of response to therapy and radiotherapy planning. When performed manually, this is a lengthy and tedious procedure which can put significant strain on clinical personnel which are often already overworked. Consequently, several automated and semi-automated methods to generate regions of interest on PET FDG have been proposed in the literature. Although the subject has received significant attention in other oncological disorders—e.g., lung cancer [27,34,45]—few works have addressed HNC. In particular, we are not aware of any previous benchmarks comparing the performance of semi-automated methods for lesion delineation of HNC lesions on FDG PET/CT. A study by Zaidi et al. [29] assessed nine methods for segmenting pharyngolaryngeal SCC on FDG PET/CT using frozen tumour specimens as a reference; while, in [25], the authors investigated the stability of radiomics features to image segmentation and signal discretisation, but the focus was on feature robustness, not on goodness of segmentation.

In this work, we compared six training-free methods for the semi-automated lesion delineation of HNC tumour regions on FDG PET/CT. With an eye on the practical implications and for reproducible research, we carried out the study using a widespread free radiomics tool (LIFEx). We found that click and draw and Nestle methods achieved the best accuracy, albeit the first at the cost of noticeable inter-patient variability and the presence of outliers towards the low-accuracy region (DSC close to zero in some cases).

We speculate that, if a voxel exhibits an unusually large uptake value, this could lead to an overestimated threshold, and, as a result, an underestimated volume—which is what happens with 40% and 50% SUVmax. The same reasoning applies to the methods of the ‘click and draw’ class. Here, the problem is that if the voxel selected as the starting point has an unusually high or low uptake, this is likely to hamper the flood-fill algorithm and produce incorrect ROIs. This could explain why click and draw and click 40 fail in some cases (see Figure 4). In contrast, the Nestle method, in which threshold calculation is mediated by background uptake, emerged as the best trade-off between the overall accuracy and robustness.

In a recent multi-centre study [21], the authors used convolutional neural networks (CNNs) to achieve an aggregate DSC of 0.774, a figure just in between that obtained here with click and draw and Nestle; whereas Andrearczyk et al. reported an average DSC of 0.717 using a bi-modal U-Net model [31]. A comparison with the recent literature therefore indicates that the accuracy attained by our two best methods was in agreement with state-of-the art approaches. We conclude observing that a fixed SUV 2.5 threshold, a commonly used approach [34,35], should be considered with care in HNC as it consistently overestimated the tumour volume by a large margin. This is likely the consequence of the ‘spillover’ effect as already discussed in [34].

The extent to which the accuracy of tumour segmentation can impact clinical practice, whether in terms of time-saving benefits or patient outcomes, is a matter for reflection. Surely one tangible advantage of computer-assisted lesion delineation is time saving, which should help streamline clinical workflows and reduce the risk of overwork. To better demonstrate the potential time benefits, we assessed the time required for manual segmentation, semi-automated ‘click and draw’ segmentation and semi-automated ‘draw and refine’ segmentation. The analysis was carried out on a sample of five cases and the results are presented in Table 4. As can be seen, the amount of time saved by semi-automated segmentation was remarkable.

In a recent work, Buteau et al. [46] investigated the influence of automated segmentation to quantify the total tumour burden (TTB) in 68Ga-PSMA-11 PET/CT scans of patients undergoing radioligand therapy (RLT) with 177Lu-PSMA. The authors concluded that automated segmentation significantly reduced the amount of time required for TTB quantification while not producing significant differences in the SUVmean. With a focus on patient outcome, a study by Li et al. [47] (MRI in colorectal cancer) demonstrated the capability of an automated segmentation tool (DeepTOP) to accurately segment tumours and predict a pathologically complete response to chemo/radiotherapy. However, large-scale clinical investigations are needed to better define the clinical relevance of semi-automated methods, especially when assisted by AI-based algorithms.

In conclusion, the main findings of the present investigation can be summarised as follows:

- Semi-automated, training-free approaches can greatly help the clinician delineate HNC lesions on FDG PET/CT;
- Among the methods considered in this study, Nestle was the best trade-off between accuracy and robustness;
- The time saving granted by semi-automated methods can be considerable;
- Fixed SUV 2.5 threshold segmentation (commonly used in the practice) should be considered with care in HNC as it consistently overestimated the tumour volume in our study.

## 5. Limitations and Future Work

There are some limitations to this work, including the relatively small sample size, the single-centre population and the retrospective nature of the study. The results should be therefore validated in larger, ideally perspective and multi-centre cohorts of patients. The scan images were used ‘as is’, therefore the potential effects of noise, filtering and/or system artefacts were considered outside the scope of the present work. Furthermore, by design, we used one single ROI as the ground-truth segmentation, hence inter-observer variability was not assessed in this study. All of the above would represent an interesting subject for future investigations.

## Figures and Tables

**Table 1:** Summary table of the methods considered in this study. Classification follows Im et al. [34].

Method	Class	Procedure	Refs.
2.5 SUV	Fixed absolute threshold	Draw and refine	[34,35]
40% SUVmax	Fixed relative threshold	Draw and refine	[17,34]
50% SUVmax	Fixed relative threshold	Draw and refine	[22,34]
Nestle	Background threshold	Draw and refine	[36]
Click and draw	Algorithm based	Click and draw	[37]
Click 40	Algorithm based	Click and draw	[37]

**Table 2:** Aggregate performance (DSC and RVD) by segmentation method. Values show median ± standard deviation. Asterisk indicates values from the literature, for a comparison; N/A stands for not available.

Segmentation Method	DSC	RVD
SUV 2.5	0.677 ± 0.172	89.9% ± 108.4
40% SUVmax	0.706 ± 0.130	−36.9% ± 100.8
50% SUVmax	0.595 ± 0.138	−56.1% ± 57.4
Nestle	0.762 ± 0.107	−32.7% ± 40.0
Click and draw	0.792 ± 0.178	−21.6% ± 1270.8
Click 40	0.701 ± 0.210	79.7% ± 2451.1
Bi-modal 3D U-Net * [31]	0.717 (mean)	N/A
3D nnU-Net * [21]	0.774 (mean)	N/A

**Table 3:** Pairwise comparison of methods (Mann–Whitney U test). Statistically significant results are marked with an asterisk followed by the direction of the inequality; $\bar{\alpha }$ indicates Bonferroni-corrected α value.

Segmentation Mode A	Segmentation Mode B	$\bar{\alpha }$	DSC	RVD
Click 40	Click and draw	0.003	<0.001 * (A < B)	<0.001 * (A > B)
Click 40	Nestle	0.003	0.004	<0.001 * (A > B)
Click 40	SUV 2.5	0.003	0.570	0.862
Click 40	40% SUVmax	0.003	0.564	<0.001 * (A > B)
Click 40	50% SUVmax	0.003	<0.001 * (A > B)	<0.001 * (A > B)
Click and draw	Nestle	0.003	0.223	0.008
Click and draw	SUV 2.5	0.003	<0.001 * (A > B)	<0.001 * (A < B)
Click and draw	40% SUVmax	0.003	<0.001 * (A > B)	<0.001 * (A > B)
Click and draw	50% SUVmax	0.003	<0.001 * (A > B)	<0.001 * (A > B)
Nestle	SUV 2.5	0.003	<0.001 * (A > B)	<0.001 * (A < B)
Nestle	40% SUVmax	0.003	0.009	0.426
Nestle	50% SUVmax	0.003	<0.001 * (A > B)	<0.001 * (A > B)
SUV 2.5	40% SUVmax	0.003	0.129	<0.001 * (A > B)
SUV 2.5	50% SUVmax	0.003	0.005	<0.001 * (A > B)
40% SUVmax	50% SUVmax	0.003	<0.001 * (A > B)	<0.001 * (A > B)

**Table 4:** Time savings: values show the total segmentation time (excluding the data loading time) for each case and by the class of segmentation method. The volume of the manually delineated ROI is also reported.

Case ID	Segmentation Time			Volume
	Manual	Click and Draw	Draw and Refine	
8229	67 s	9 s	18 s	3.0 cm${}^{3}$
16f6	75 s	8 s	14 s	2.3 cm${}^{3}$
a174	92 s	9 s	13 s	6.0 cm${}^{3}$
58b2	88 s	11 s	21 s	12.2 cm${}^{3}$
b6d2	308 s	11 s	22 s	38.3 cm${}^{3}$
Avg	126.0 s	9.6 s	17.6 s	

## Data Availability

Anonymised by-patient results are provided as supplementary material (anonymised_by_patient_accuracy.csv). Original FDG PET/CT scans are not available due to privacy restrictions.

## Supplementary Materials

The following supporting information can be downloaded at: https://www.mdpi.com/article/10.3390/s23187952/s1, Table S1: By-patient results.

## Abbreviations

The following abbreviations are used in this manuscript:

CNN	Convolutional neural network(s)
EBV	Epstein–Barr virus
FDG PET/CT	Positron emission tomography/computed tomography with [${}^{18}$F] fluorodeoxyglucose
HNSCC	Head and neck squamous cell carcinoma
HNC	Head and neck cancer
HPV	Human papillomavirus
MRI	Magnetic resonance imaging
PSMA	Prostate-specific membrane antigen
ROI	Region(s) of interest
SUV	Standardised uptake value
TTB	Total tumour burden
